# A bibliometric analysis of global research status and trends in neuromodulation techniques in the treatment of autism spectrum disorder

**DOI:** 10.1186/s12888-023-04666-3

**Published:** 2023-03-20

**Authors:** Lifei Xiao, Xianhao Huo, Yangyang Wang, Wenchao Li, Mei Li, Chaofan Wang, Feng Wang, Tao Sun

**Affiliations:** 1 Department of Neurosurgery, General Hospital of Ningxia Medical University, Yinchuan, 750000 China; 2 Ningxia Key Laboratory of Cerebrocranial Disease, Incubation Base of National Key Laboratory, Ningxia Medical University, Yinchuan, 750000 China; 3 Department of Neurosurgery, The First Affiliated Hospital, Zhejiang University School of Medicine, Hangzhou, 310000 China

**Keywords:** Autism spectrum disorder, Neuromodulation techniques, Bibliometrics, Visualization analysis, Transcranial magnetic stimulation, Transcranial direct current stimulation

## Abstract

**Background:**

Autism spectrum disorder (ASD) is a neurodevelopmental disease which has risen to become the main cause of childhood disability, placing a heavy burden on families and society. To date, the treatment of patients with ASD remains a complicated problem, for which neuromodulation techniques are a promising solution. This study analyzed the global research situation of neuromodulation techniques in the treatment of ASD from 1992 to 2022, aiming to explore the global research status and frontier trends in this field.

**Methods:**

The Web of Science (WoS) was searched for literature related to neuromodulation techniques for ASD from 1992 to October 2022. A knowledge atlas to analyze collaboration among countries, institutions, authors, publishing journals, reference co-citation patterns, keyword co-occurrence, keyword clustering, and burst keywords was constructed using Rstudio software, CiteSpace, and VOSviewer.

**Results:**

In total, 392 publications related to the treatment of ASD using neuromodulation techniques were included. Despite some fluctuations, the number of publications in this field has shown a growing trend in recent years. The United States and Deakin University are the leading country and institution in this field, respectively. The greatest contributing authors are Peter G Enticott, Manuel F Casanova, and Paul B Fitzgerald et al. The most prolific and cited journal is *Brain Stimulation* and the most commonly co-cited journal is *The Journal of Autism and Developmental Disorders*. The most frequently cited article was that of Simone Rossi (Safety, ethical considerations, and application guidelines for the use of transverse magnetic stimulation in clinical practice and research, 2009). “Obsessive–compulsive disorder,” “transcranial direct current stimulation,” “working memory,” “double blind” and “adolescent” were identified as hotspots and frontier trends of neuromodulation techniques in the treatment of ASD.

**Conclusion:**

The application of neuromodulation techniques for ASD has attracted the attention of researchers worldwide. Restoring the social ability and improving the comorbid symptoms in autistic children and adults have always been the focus of research. Neuromodulation techniques have demonstrated significant advantages and effects on these issues. Transcranial magnetic stimulation (TMS) and transcranial direct current stimulation (tDCS) are new therapeutic methods introduced in recent years, and are also directions for further exploration.

**Supplementary Information:**

The online version contains supplementary material available at 10.1186/s12888-023-04666-3.

## Introduction

Autism spectrum disorder (ASD) is a group of neurodevelopmental disorders characterized by social communication impairment and restricted interests/repetitive behaviors, which mainly occur in childhood and affect their whole life [1]. A recent systematic analysis of the global status of mental disorders showed that the global age-standardized prevalence of ASD was 369.4 cases per 100,000 people in 2019, with a significant male predominance [2]. ASD is often an overwhelming experience for parents, placing a heavy burden on families and society, which is reflected in both mental and economic burdens [3, 4]. A Previous study have shown that the economic cost of ASD mainly stems from special education and parental productivity loss in childhood and supportive living accommodation and individual production loss in adults [4]. In addition, medical costs are much higher for adults with ASD than for children. In the United States and the United Kingdom, the cost of supporting autistic patients without intellectual disabilities is approximately $ 1.4 million, while the cost of supporting autistic patients with intellectual disabilities is higher, reaching $ 2.4 million [5]. Although the pathogenesis of ASD remains poorly understood, current research suggests that it may be related to both genetic inheritance [6] and environmental factors [7]. The cellular etiology of ASD includes abnormalities of one or more developmental events, including neurogenesis, neuronal migration, axonal projection, dendritic development, synaptogenesis and synaptic remodeling [8]. Among them, the pioneering research of Rubenstein and Merzenich shows that the imbalance between excitatory and inhibitory neurosignaling (E/I imbalance) is a potential neuropathophysiology of autism, which is mainly due to the enhancement of glutamate (excitatory) signal pathway, or the reduction of inhibition caused by the weakening of GABAergic signal pathway [9]. The migration of neurons in the cortex is mainly controlled by the paracrine action of neurotransmitters glutamate and GABA [10], E/I imbalance can lead to abnormal differentiation and migration of neuron, delayed synapse maturation or abnormal myelination [9]. The defect of neuronal migration also leads to the mis-localization of affected neurons [8], and the consequent malformations and malfunctions of various brain circuits are considered to be the important causes of various neurodevelopmental disorders, including epilepsy [11, 12], intellectual impairment [13] and ASD [12, 14, 15]. Abnormal changes in the levels of GABA receptors and GAD65/67 in brain samples of austic patients were also confirmed in animal models [16–21]. Defects in either the production or migration of GABAergic neurons in the cortex led to the decrease of the number of GABAergic neurons in the cortex, resulting in the over-excitation of the cortex, thus affecting the synaptic plasticity process [22]. In addition, neural plasticity can also be mediated by secondary neurotransmitters transmitted by subcortical nucleus, such as serotonin, acetylcholine, dopamine [23–26]. In short, the important theory of E/I imbalance provides direction and basis for the later research on the treatment of ASD. Early psychological/behavioral intervention, complementary therapy, or drug treatment can improve some symptoms of autism, such as reducing anxiety and aggression, reducing the combined symptoms of attention deficit and hyperactivity, and promoting emotional communication [1]. However, these treatments cannot completely correct the core symptoms of ASD, such as social communication impairment, which leads to many problems, such as poor patient tolerance, adverse reactions, and economic burdens [27]. Therefore, novel therapeutic methods are required.

In the past decade, neuromodulation techniques have made impressive progress in the treatment of neuropsychiatric diseases [28, 29]. Deep brain stimulation (DBS) has been widely clinically applied to effectively improve the symptoms of Parkinson's disease, such as tremor, slowed movement, and rigid muscles [30]. Preclinical studies have shown that DBS in the prefrontal cortex, hypothalamic nucleus, and central thalamus can alleviate VPA (Valproate acid) -induced autism-like behaviors [31–33]. Vagus nerve stimulation (VNS) is an FDA (Food and Drug Administration) -approved therapy to reduce the severity of intractable epilepsy and depression, which has become a potential adjuvant therapy for patients with autism [34]. Dysregulated parasympathetic system and reduced vagal tone are frequently observed in ASDs, which is related to autistic behavioral and language disorders [35, 36]. VNS treatment has been proven to overcome the problem of an insufficient vagal response [37], suggesting that VNS treatment may be beneficial for neurodevelopmental disorders with altered parasympathetic activity. In addition, research on the use of VNS in children with epilepsy and ASD has achieved positive results [38]. In recent years, promising results in adult neuropsychiatric diseases have promoted active research on noninvasive neuromodulation techniques in children and adolescents, particularly transcranial magnetic stimulation (TMS) and transcranial direct current stimulation (tDCS) [39]. The stimulation frequency and duration of stimulation period determine the effects caused by rTMS [40]. Low-frequency stimulation (< 1 Hz) has inhibitory effects, whereas high-frequency stimulation (> 5 Hz) leads to excitatory effects in the brain [41]. In addition, low intensity (less than motor threshold) tends to reduce cortical excitability, while high intensity (greater than motor threshold) will increase cortical excitability [42]. Early research of ASD mainly focused on the conventional low-frequency (1 Hz) repetitive TMS (rTMS) in the prefrontal cortex, and proved its positive impact on the behavior and electrophysiological results of children with ASD [43, 44]. In recent years, high-frequency theta-burst stimulation protocols to the motor cortex was also tested in children and adults [45, 46]. Previous studies have shown that rTMS can affect cortical excitability by altering the glutamatergic (Glx) or γ-aminobutyric acid (GABA) metabolite levels [47], and reducing irritability, hyperactivity, and stereotyped behavior in autistic children [43, 48, 49]. In addition, it also has a positive impact on children’s error monitoring and correction function [50]. Transcranial direct current stimulation (tDCS) is considered to be a promising method for the rehabilitation of neurodevelopmental disorders [51]. In humans, the fast oscillation of electroencephalogram (EEG) activity occurs in the frequency range of 30-120 Hz, which is called “gamma” frequency band [52]. Several studies showed that gamma band response (30-80 Hz) is very dependent on E/I signal transduction [53–55], mediating a series of basic neural functions, including sensorimotor integration, perceptual integration, working memory, network synchronization and higher-order cognition [52, 56–58], which is obviously disturbed in multiple systems of ASD [59–61]. A recent EEG study for adults found that GABA was coupled with gamma band response, and reported a positive correlation between GABA level and gamma band power [62]. tDCS uses constant weak current to induce bidirectional and polar dependent changes in the cortex, which has been proved to regulate gamma oscillation and excitability in the cortex [63, 64]. Recent clinical research shown that the tDCS in the prefrontal cortex, motor cortex and temporoparietal junction regions can effectively improve the social function and abilities of the theory of mind in patients with ASD [65–68].

Neuromodulation techniques are rapidly changing the field of neuroscience, especially in recent years, and research on ASD has surged, showing that it is necessary to conduct a comprehensive analysis of the current situation and trends. Bibliometrics is widely used to analyze the published literature, and is an effective way to understand the development of the discipline. In recent years, bibliometric analysis has been conducted on the status and research trends of brain tumors [69], neurodegenerative diseases [70], and neuropsychiatric diseases [71]. However, to the best of our knowledge, there has been no bibliometric analysis investigating the application of neuromodulation techniques in ASD. Therefore, in the present study, we analyzed the global research status of neuromodulation techniques for the treatment of ASD from 1992 to October 2022 using bibliometrics and literature visualization tools. The results are presented in the form of a visual map to further analyze the research hotspots, future trends, and application prospects of neuromodulation techniques in ASD.

## Methods

### Search strategy

**Fig. 1:**
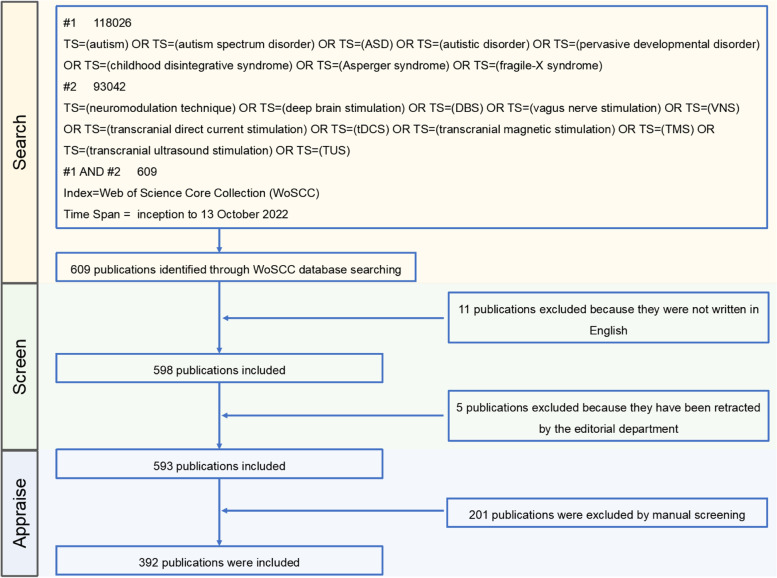
Flow chart of the literature screen

Full records of all relevant publications were obtained from the Web of Science (WoS) on October 13, 2022. The main topics of data retrieval were “neuromodulation techniques” and “autism spectrum disorder”. Although the most recent diagnostic criteria for “autism spectrum disorder” in the Diagnostic and Statistical Manual of Mental Disorders (Fifth Edition) (DSM-5) is broader and no longer subdivided into “pervasive developmental disorder not otherwise specified,” “autistic disorder,” and “Asperger syndrome,” these medical terms are still widely used in clinical practice or research [72, 73]. As such, the search strategy was described as follows: TS = (“autism” OR TS = “autism spectrum disorder” OR TS = “ASD” OR TS = “autistic disorder” OR TS = “pervasive developmental disorder” OR TS = “childhood disintegrative syndrome” OR TS = “Asperger syndrome” OR TS = “fragile-X syndrome”) AND TS = (“neuromodulation techniques” OR TS = “deep brain stimulation” OR TS = “DBS” OR TS = “vagus nerve stimulation” OR TS = “VNS” OR TS = “transcranial direct current stimulation” OR TS = “tDCS” OR TS = “transcranial magnetic stimulation” OR TS = “TMS” OR TS = “transcranial ultrasound stimulation” OR TS = “TUS”). The publication date of all documents ranged from January 1, 1992, to October 31, 2022. In the literature search phase, the data were independently collected by two authors (LFX and YYW) before comparison; literature with differences was retained or excluded after discussion. Literature retrieval was not limited to the category or type of article, but the publication language was limited to English. In the manual screening phase, only literature involving neuromodulation techniques in the treatment of ASD was included, and duplicate studies were removed. Any disagreements were discussed or by seeking the help of other authors. Finally, out of 609 studies identified in the initial search, 392 met the screening criteria and were included for further analysis. Software analysis was performed independently by WCL. The retrieval process is illustrated in Fig. 1.

### Statistical analysis

Data from publications screened from the Web of Science were analyzed using the bibliometrix package of the Rstudio software (version 1.4.1717) and Citespace (version5.6. r4), and Vosviewer (Version1.6.14). The “social structure function” in bibliometrix package was used to plot a collaboration world map of different countries. Citation space is a type of citation visualization analysis software developed gradually against the background of scientometrics and data visualization. The structure, rules, and distribution of scientific knowledge are presented visually, so as to constitute a “scientific knowledge map,” which reflects the progress and the current research frontiers of a certain field. VOSviewer is a software tool for building and visualizing bibliometric networks that can be constructed based on citations, bibliographic coupling, common citations, or co-authorship relationships. In the map created by Vosviewer, each node represents an element such as a country, institution, or author. In the present study, CiteSpace was used to analyze the clustering and timeline of keywords, as well as the centrality of the country, institution, author, and keywords. Vosviewer was used to analyze the clustering of countries, institutions, authors, journals, and keywords, including the number of publications, citations, and the link strength of each element. To maintain consistency, we set the number threshold of each node to three, so that only elements with a number (such as the number of publications) greater than three is displayed in the graph. The larger the link width between the nodes, the stronger the degree of cooperation, and the larger the size of the nodes, the greater the number of reflections.

## Results

### Analysis of quantity and annual trend of published literature

**Fig. 2:**
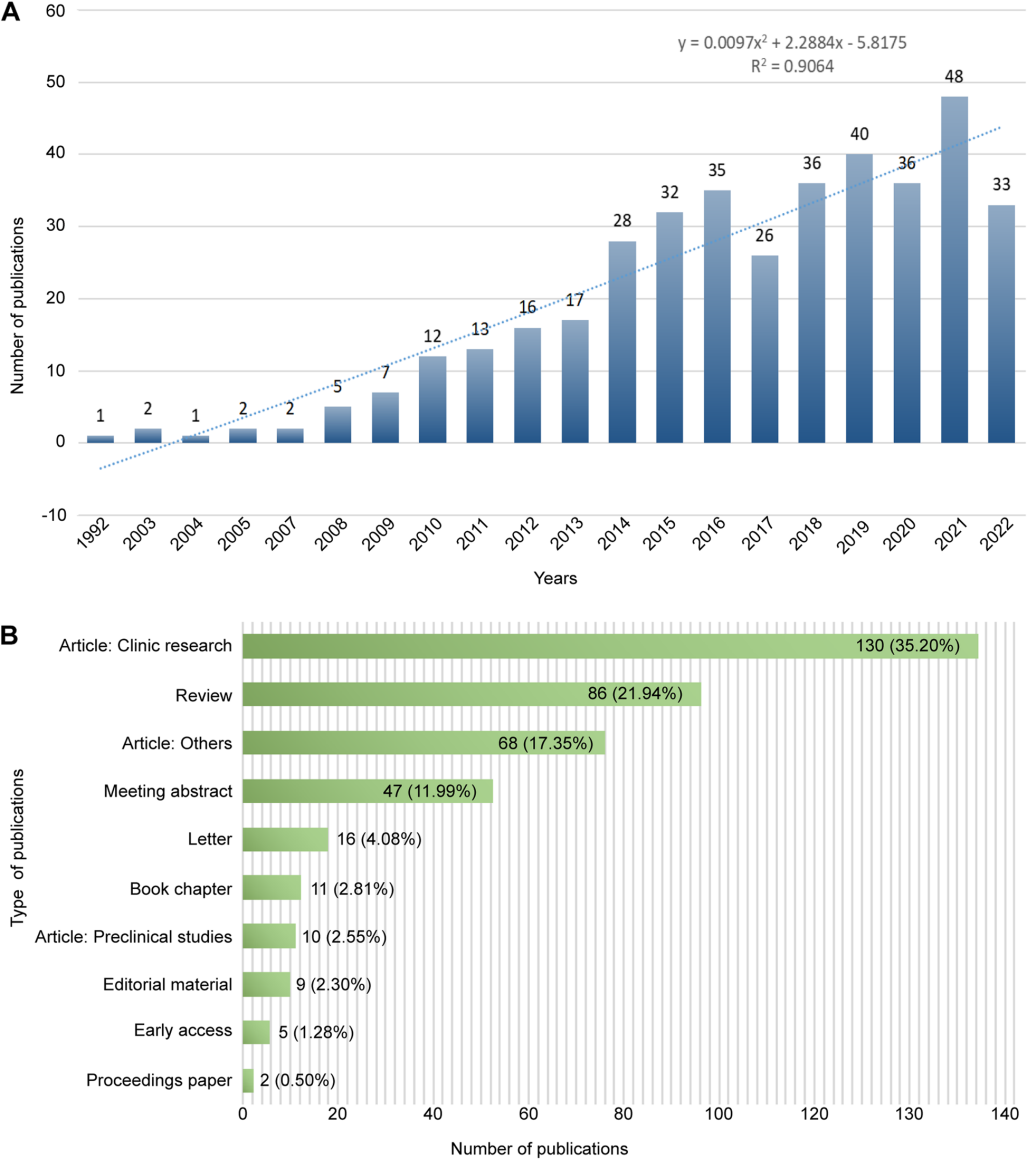
Analysis of Publications. **A** Annual trends in publications related to neuromodulation techniques for ASD from inception to 13 October 2022. **B** The type and number of publications

A total of 609 articles were retrieved from WoS, and those unrelated to neuromodulation techniques for the treatment of ASD or repeated were excluded. In total, 392 articles were published between 1992 and October 2022. The number of publications published in 2021 was 48, the highest in recent years. Although there were some fluctuations in the number of studies on neuromodulation techniques for ASD between 2017 and 2020, polynomial model fitting (R^2^ = 0.9064) predicted a significant correlation between publication year and publication yield, suggesting that the number of studies in this field will continue to increase in the future, indicating that the application of neuromodulation techniques in ASD has become a current frontier field. The related results are shown in Fig. 2A.

### Document type analysis

Eight literature types were screened (Fig. 2B). Article was the most frequently published category of literature, accounting for 55.10% of the total literature, of which the majority were clinical studies (138, 35.20%), including randomized controlled trials, cohort studies, and case reports, which mainly investigated the role of noninvasive neuromodulation techniques in the treatment of ASD, such as TMS and tDCS. This was followed by other types of research (68, 17.35%), such as methodology, meta-analysis, and review, which were included in the journal as articles. Finally, preclinical studies (10, 2.55%) mainly involved DBS in rodent models of ASD. Review was the second largest category of literature type (86, 21.94%), and meeting abstracts was the third largest literature type (47, 11.99%).

### Country production analysis

**Fig. 3:**
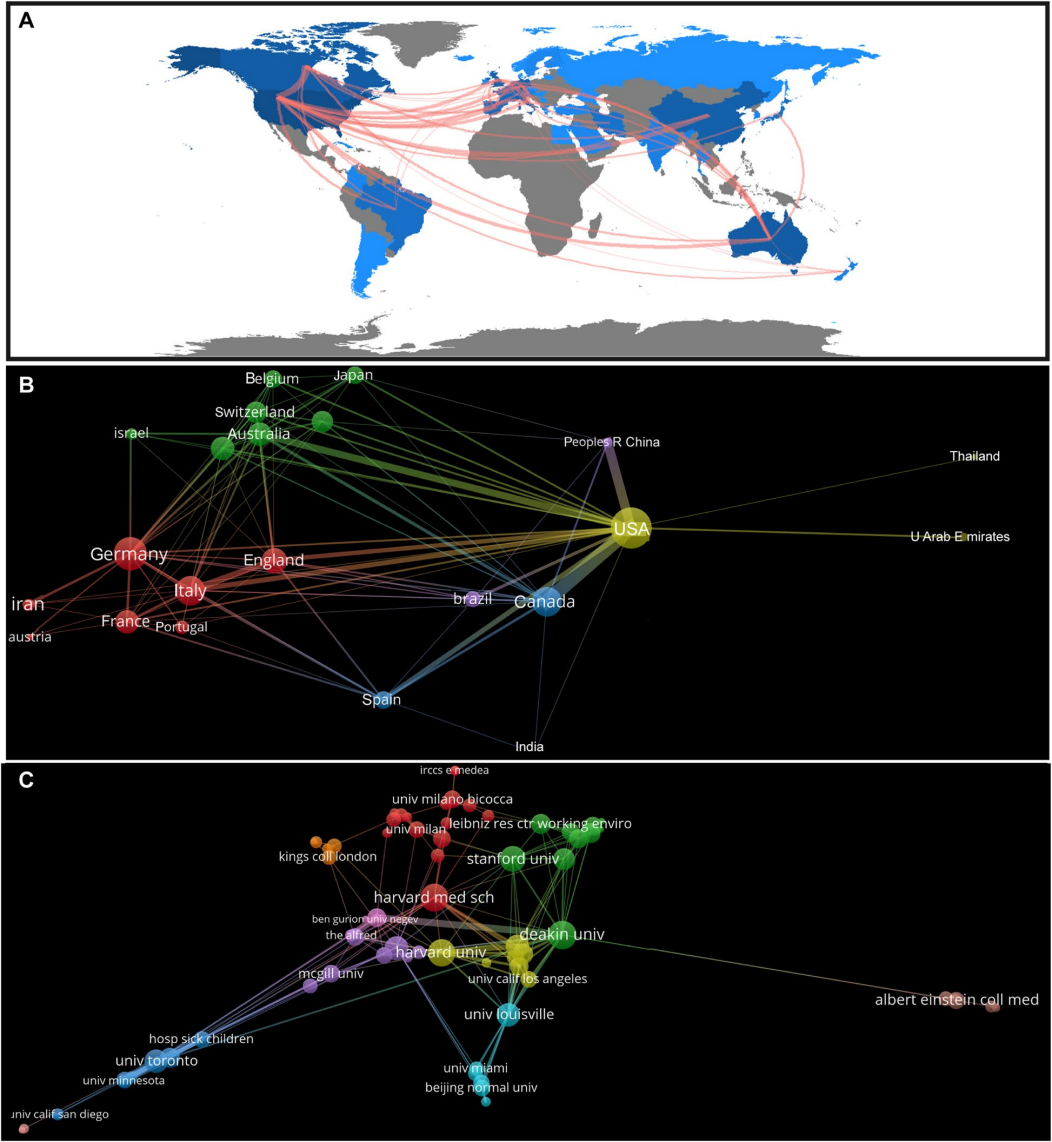
Analysis of countries and institutions. **A** The collaboration network shown on the world map. Darker blue indicates higher collaboration rates, the wider the link line, the higher the rate of collaboration between the two countries. **B** Analysis of collaboration among countries with more than three publications. **C** Analysis of collaboration among institutions with more than three publications. Clusters were identified by color, the size of the circles represent the number of publications, and the thickness of the lines shows the strength of the association

A total of 46 countries spanning Asia, Europe, North America, South America, and Oceania, were found to be involved in neuromodulation research for ASD, showing a global collaboration trends (Fig. 3A). It is worth noting that research in this field is mainly concentrated in developed countries, with European countries having the most research. Some top economies around the world, such as China and Iran, are also involved in research in this area. The top three countries in terms of the number of publications are the USA (172), Canada (50), and Australia (49). The USA was the most frequently cited country (4,247), followed by Australia (1,693) and Italy (1,264). The top three countries in terms of total link strength were USA (91), Canada (48), and Italy (40), which reflects the degree of international cooperation in this field. In addition, the top three countries in terms of centrality are the USA (0.39), the United Kingdom (0.27), and Canada (0.14), which indicates the authority and degree of concern of these countries in this field (Table 1). The USA has the highest number of publications, citations, link strength, and centrality, which shows that research results in the treatment of ASD with neuromodulation techniques in the USA have attracted extensive attention.

**Table 1:** Top 10 publications, citations, total link strength and centrality of countries related to neuromodulatin techniques on ASD

Rank	Publications	Citations	Total link strength	Centrality
1	USA (172)	USA (4247)	USA (91)	USA (0.39)
2	Canada (50)	Australia (1693)	Canada (48)	England (0.27)
3	Australia (49)	Italy (1264)	Italy (40)	Canada (0.14)
4	England (36)	England (1212)	Germany (38)	Italy (0.13)
5	Italy (33)	Canada (1093)	Australia (36)	Germany (0.13)
6	Germany (32)	Germany (1009)	England (35)	Israel (0.07)
7	China (32)	Netherlands (604)	Spain (26)	Spain (0.05)
8	Spain (19)	Israel (502)	China (20)	Australia (0.04)
9	France (17)	France (404)	France (19)	France (0.03)
10	Netherlands (14)	China (335)	Netherlands (16)	Netherlands (0.01)

The cooperative analysis map shows the cooperative situation of the main countries; 23 countries with more than three publications were displayed by the Vosviewer software (Fig. 3B). The USA and Canada have the highest intensity of cooperation, while the USA, China, Australia, Italy, Spain, and the United Kingdom also maintain a high intensity of cooperation, which constitutes the most important multi-center cooperation network in this field. Taking the top three clusters as an example, yellow clustering shows cooperation among the USA, the United Arab Emirates, and Thailand; red clustering shows cooperation among Germany, Italy, the United Kingdom, France, etc.; and green clustering shows cooperation among Australia, the Netherlands, New Zealand, Switzerland, Belgium, and Japan. In recent years, publications on the treatment of ASD with neuromodulation techniques mainly came from Belgium, Portugal, and China, which reflects the active degree of these countries in this field (Supplementary Fig. 1A).

### Institutional analysis

A total of 633 institutions are involved in research on neuromodulation techniques for the treatment of ASD. Deakin University (24) has the largest number of publications, followed by the University of Louisville (21), and the University of Toronto (21). Harvard University (893) was the most frequently cited institution, followed by Monash University (656), and Louisville University (598). The top three institutions in terms of total link strength are Deakin University (32), Beth Israel Deaconess Medical Center (31), and University of Toronto (28), indicating the degree of inter-institutional cooperation in this field. In addition, the top three institutions of centrality are Deakin University (0.12), Harvard University (0.12), and Harvard Medical School (0.09), indicating the authority and degree of concern of these institutions in this field (Table 2).

**Table 2:** Top 10 publications, citations, total link strength and centrality of institutions related to neuromodulation techniques on ASD

Rank	Publications	Citations	Total link strength	Centrality
1	Deakin University (24)	Harvard University (893)	Deakin University (32)	Deakin University (0.12)
2	University of Louisville (21)	Monash University (656)	Beth Israel Deaconess Medical Center (31)	Harvard University (0.12)
3	University of Toronto (21)	University of Louisville (598)	University of Toronto (28)	Harvard Medical School (0.09)
4	Monash University (20)	Harvard Medical School (461)	Harvard University (26)	Monash University (0.08)
5	Harvard University (18)	Deakin University (434)	Mayo Clinic (24)	University of Toronto (0.07)
6	Centre for Addiction and Mental Health (17)	Beth Israel Deaconess Medical Center (413)	Centre for Addiction and Mental Health (24)	University of Louisville (0.04)
7	Harvard Medical School (17)	The Alfred (352)	Brown University (23)	Beth Israel Deaconess Medical Center (0.04)
8	Mayo Clinic (16)	The University of Queensland (342)	University of Louisville (22)	Massachusetts General Hospital (0.04)
9	Beth Israel Deaconess Medical Center (12)	University of Toronto (336)	Monash University (21)	University of Sao Paulo (0.04)
10	The Hospital for Sick Children (10)	University College London (262)	Harvard Medical School (20)	Beijing Normal University (0.03)

The cooperative analysis map shows the main institutional cooperative situation, for which 75 institutions with more than three publications are displayed (Fig. 3C). Taking the top three clusters as an example, green clustering shows cooperation among Deakin University, Stanford University, Leibniz Research Centre for Working Environment and Human Factors and Columbia University, etc., red clustering shows cooperation among Harvard Medical School, University of Cambridge, Milan Bicocca University and University of Milan, etc., yellow clustering shows cooperation among Harvard University, Beth Israel Deaconess Medical Center, Brown University, and Boston Children's Hospital, etc. Deakin University, the University of Louisville, Monash University, the University of Toronto, Harvard University, and Harvard Medical School were the key nodes in the collaboration network, which is characterized by small-scale aggregation and extensive cooperation, promoting the globalization of research in this field (Fig. 3C). In recent years, the more active institutions in the research on the treatment of autism with neuromodulation techniques are the Hospital for Sick Children, University of Sao Paulo, and University of South Carolina. Research from these institutions has mainly focused on the clinical application or theory of TMS and tDCS in children, adolescents, and adults with ASD [74–77] (Supplementary Fig. 1B).

### Author analysis

A total of 1,596 authors have published literature on the treatment of ASD using neuromodulation techniques, 15 of whom have published more than 10 articles. Table 4 lists the top 10 most prolific authors. The top three authors with the largest number of publications were Peter G Enticott (28), Manuel F Casanova (26), and Paul B Fitzgerald (18). The most frequently cited authors were Peter G Enticott (734), Paul B Fitzgerald (664), and Alvaro Pascual Leone (656). The top three authors in terms of total link strength, which reflects the degree of cooperation between authors in this field, are Manuel F Casanova (97), Zafiris J Daskalakis (97), and Pushpal Desarkar (86). The top three authors in terms of centrality are Peter G Enticott (0.06), Manuel F Casanova (0.03), and Paul B Fitzgerald (0.03), which indicates the authority and degree of concern of these authors in this field (Table 3). Interestingly, we observed extensive cooperation among the top ten authors, who are co-authors of many studies, such as Peter G Enticott, Manuel F Casanova, Paul B Fitzgerald, Estate M Sokhadze, Alvaro Pascal Leone, Lindsay M Oberman, and Lonnie L Sears.

**Table 3:** Top 10 publications, citations, total link strength and centrality of authors related to neuromodulation techniques on ASD

Rank	Publications	Citations	Total link strength	Centrality
1	Enticott PG (28)	Enticott PG (734)	Casanova MF (97)	Enticott PG (0.06)
2	Casanova MF (26)	Fitzgerald PB (664)	Daskalakis ZJ (97)	Casanova MF (0.03)
3	Fitzgerald PB (18)	Pascual-Leone A (656)	Desarkar P (86)	Fitzgerald PB (0.03)
4	Sokhadze EM (18)	Casanova MF (584)	Blumberger DM (84)	Oberman LM (0.03)
5	Pascual-Leone A (14)	Rinehart NJ (392)	Enticott PG (82)	Daskalakis ZJ (0.03)
6	Oberman LM (14)	Oberman LM (374)	Lai MC (82)	Croarkin PE (0.02)
7	Sears L (14)	Sokhadze EM (370)	Croarkin PE (78)	Blumberger DM (0.01)
8	Daskalakis ZJ (14)	Sears L(352)	Szatmari P (74)	Pascual-Leone A (0.01)
9	Croarkin PE (14)	Bradshaw JL (287)	Sokhadze EM (66)	Sokhadze EM (0.01)
10	Desarkar P (12)	Kennedy HA(286)	Ameis SH (65)	Ameis SH (0.01)

**Fig. 4:**
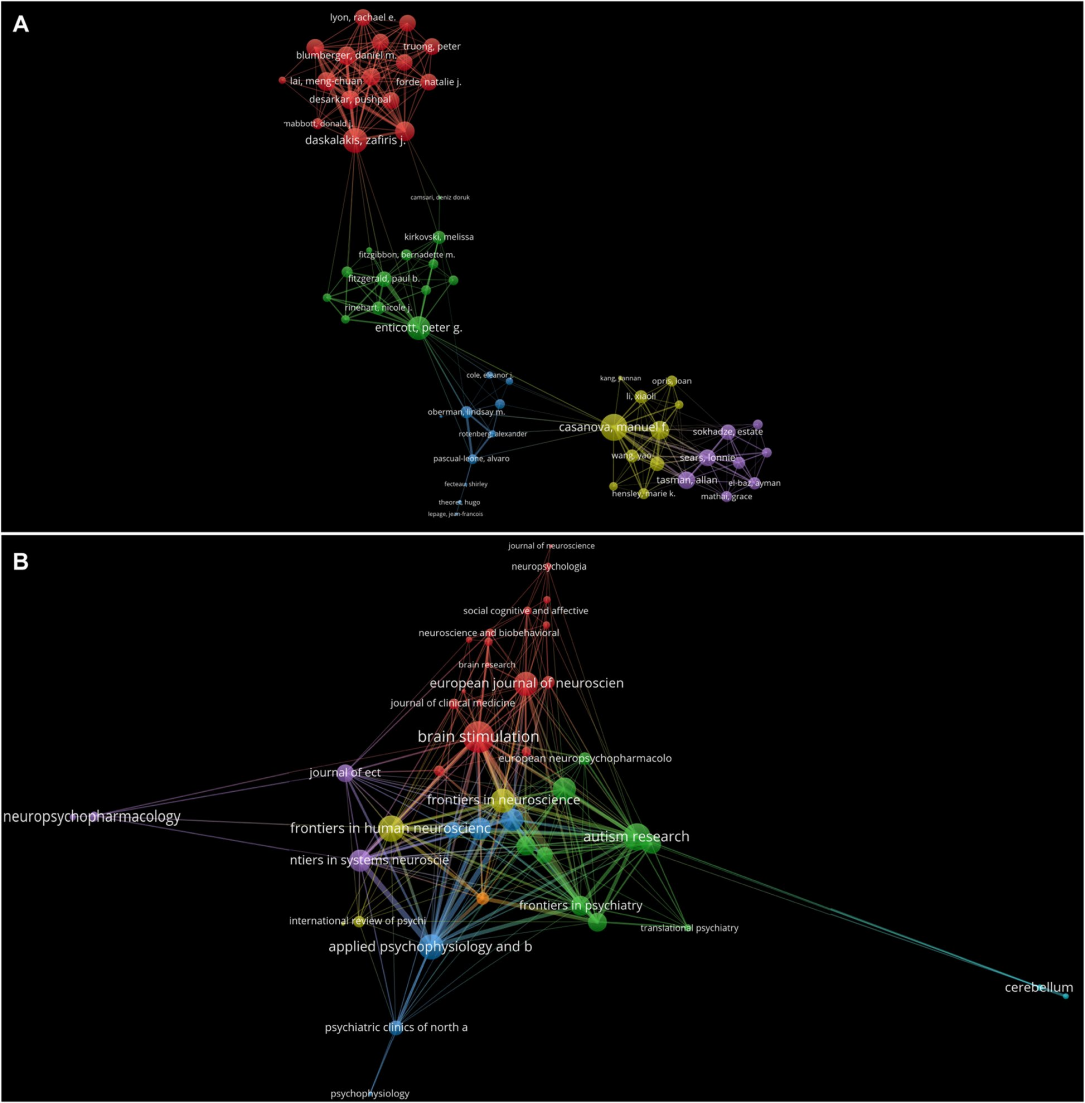
Analysis of authors and journals. **A** Analysis of co-authorship among authors with more than three publications. **B** Visualization of the published journal network with more than three publications

The cooperative analysis map shows the authors’ cooperative situation, and 91 authors with more than three publications are displayed (Fig. 4A). There were five clusters in total, and the top three clusters were considered as examples. The red cluster includes the authors Zafiris J Daskalakis, Paul E Croarkin, Pushpal Desarkar, Daniel M Blumberger, Stephanie H Ameis, and Meng-Chuan Lai, et al., whose research focused on the effect of TMS on neurotransmitters in young ASD patients [47, 76, 78]. Enticott, Fitzgerald, Rinehart, and Bradshaw are the predominant members of the green cluster. They mainly studied the effects of TMS on the inhibition and excitability of the cerebral cortex in patients with autism [49, 79, 80]. Yellow clustering represents Manuel F Casanova, Estate M Sokhadze, Ayman S El Baz, Yao Wang, and Xiaoli Li et al., whose research mainly focused on the effects of TMS on neurofeedback in children with autism [81–83] (Fig. 4A). As shown in Supplementary Fig. 2A, Zafiris J Daskalakis, Paul E Croarkin, Pushpal Desarkar, Daniel M Blumberger, Stephanie H Ameis, and Meng Chuan Lai have been active in the field of neuromodulation techniques for the treatment of autism in recent years, and their research continues on the application of TMS in autism.

### Analysis of contributions of journals/conferences

A total of 183 journals have published literature on the treatment of autism with neuromodulation techniques, ten of which published more than five studies. *Brain Stimulation* was the most published journal, with 15 articles, followed by *Applied Psychophysiology and Biofeedback* (15) and *Frontiers in Neuroscience* (11). In terms of cited frequency, *Brain Stimulation* ranked first (425), followed by *The Cerebellum* (379), and *Biological Psychiatry* (295) (Table 4). Figure 4B shows the 44 journals with more than three studies in this field. Red clustering includes *Brain Stimulation, Neuroscience & Biological Reviews,* and *European Journal of Neuroscience*. According to the literature published in recent years, these journals are more inclined to accept studies related to neuron-modulation techniques in clinical practice, such as its impact on autistic behavior [68]. Green clustering includes *Biological Psychiatry, Frontiers in Psychiatry,* and *Autism Research*. These studies have mainly focused on the potential mechanisms of neuromodulation techniques in the treatment of autism [48]. Blue clustering includes *Applied Psychophysiology and Biofeedback, The Journal of Autism and Developmental Disorders,* and *Autism*. These journals cover a wide range of disciplines, but have mainly published articles related to the impact of neuromodulation techniques on neurofeedback in this field [83]. In recent years, journals such as *Frontiers in Psychiatry* and *Autism Research* have been active in this field, publishing articles involving TMS or tDCS randomized controlled trials in patients with ASD (Supplementary Fig. 2B). In addition, *Journal of Autism and Developmental Disorders* (833), *Brain Stimulation* (699) and *Neuroimage* (677) are the most frequently co-cited journals (Table 5).

**Table 4:** Top 10 journals related to neuromodulation techniques on ASD

Rank	Publications	Citation
1	Brain Stimul (15)	Brain Stimul (425)
2	Appl Psychophysiol Biofeedback (15)	Cerebellum (379)
3	Front Neurosci (11)	Biol Psychiatry (295)
4	Front Hum Neurosci (10)	Cortex (275)
5	Biol Psychiatry (9)	Front Hum Neurosci (269)
6	Front Psychiatry (9)	Eur J Neurocsi (224)
7	Autism Res (9)	Appl Psychophysiol Biofeedback (211)
8	Neuropsychopharmacology (8)	Neurosci Biobehav Rev (208)
9	Neurosci Biobehav Rev (7)	Neuropsychopharmacology (203)
10	Eur J Neurocsi (6)	Front Neurosci (191)

**Table 5:** Top 10 co-cited journals related to neuromodulation techniques on ASD

Rank	Journal	Citation
1	J Autism Dev Disord	833
2	Brain Stimul	699
3	Neuroimage	677
4	Biol Psychiat	641
5	J Neurosci	633
6	Clin Neurophysiol	542
7	Neuron	432
8	Brain	387
9	Nature	358
10	P Natl Acad Sci USA	358

**Fig. 5:**
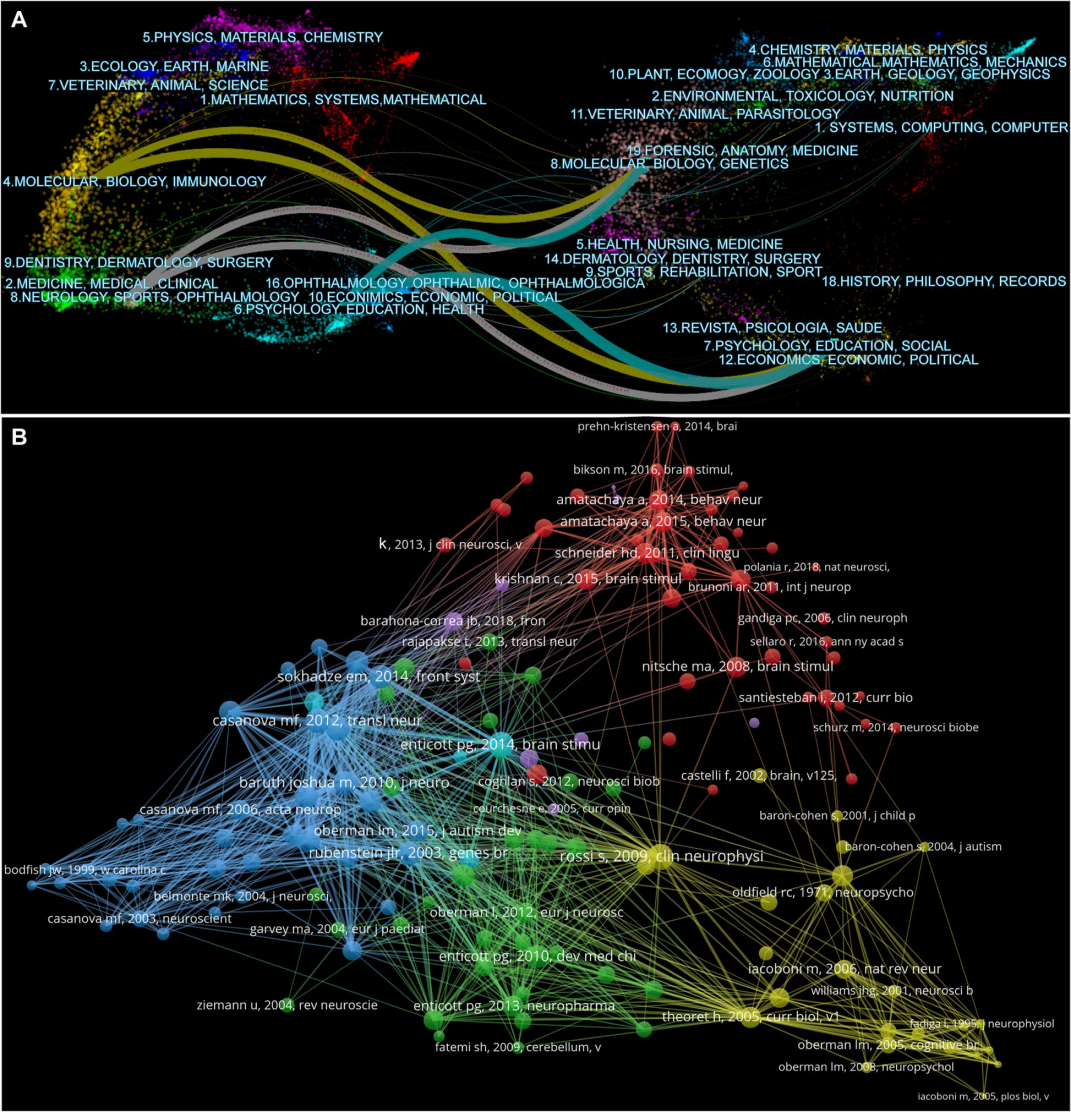
Analysis of cited and citing journals. **A** The dual-map overlays of cited journals and the citing journals. **B** Visualization of the co-cited references network

Figure 5A illustrates the reciprocal relationship between cited and citing journals. We found that journals related to neurology, psychology, education, and health are obviously related to molecular, biological, genetic and social fields, illustrating that researchers attempted to uncover the relationship between specific molecular or biological targets such as genes and psychological/social disorders of ASD patients [47, 83, 84]. In addition, there are extensive reciprocal references between journals of the same type, such as psychology, education, molecules, and biology.

Although the meeting abstracts and conference proceedings lack details of the relevant studies, they remain an important source to understand the progress of research in this field. The American College of Neuropsychopharmacology (ACNP), Society of Biological Psychiatry (SOBP), and Congress of the European College of Neuropsychopharmacology (ECNP) are major sponsors of international conferences. From 2003 to 2022, 42 international conferences related to the target topic were held. In this field, conferences mainly discussed the treatment of ASD with epilepsy by VNS [85], the application of DBS in Fragile X-associated tremor/ataxia syndrome [86], and the treatment of autistic self-injurious behavior by DBS [87]. Since 2014, the focus of research has shifted to non-invasive neuromodulation techniques for treating patients with ASD, especially TMS and tDCS [88–90].

### Reference analysis

A total of 20,182 references were co-cited in the selected publications, of which 154 were co-cited more than 10 times (Fig. 5B). The top ten co-cited references are listed in Table 6. Rossi et al. had the highest citation counts (56), followed by Enticott et al. (53), and J L R Rubenstein et al. (48). Rossi et al. predominantly discussed the safety, ethical considerations, and application guidelines in clinical practice and research on TMS, It provides certain reference and guidance value for the follow-up clinical application of TMS [91]. Enticott et al. discussed the impact of TMS in the bilateral dorsomedial prefrontal cortex on the social function of ASD patients, which to some extent expanded the stimulation target of TMS and its applicability in mental diseases [92]. J L R Rubenstein et al. paid more attention to the alteration of molecules or biological functions in ASD, such as the abnormal ratio of excitation/inhibition in key neural systems, which provides clues for the potential underlying mechanisms or targets of autism treatment [9]. It is worth noting that seven of the ten most co-cited studies discussed the impact of TMS on ASD patients, reflecting to some extent the importance and popularity of TMS in this field (Table 6).

**Table 6:** Top 10 co-cited references related to neuromodulation techniques on ASD

Rank	Cited reference	citations	The first author/corresponding author (publication year)	Journal
1	Safety, ethical considerations, and application guidelines for the use of transcranial magnetic stimulation in clinical practice and research	56	Rossi S (2009) [91]	Clin Neurophysiol
2	A double-blind, randomized trial of deep repetitive transcranial magnetic stimulation (rTMS) for autism spectrum disorder	53	Enticott PG (2014) [92]	Brain Stimul
3	Model of autism: increased ratio of excitation/inhibition in key neural systems	48	Rubenstein JL (2003) [9]	Genes Brain Behav
4	Low-frequency repetitive transcranial magnetic stimulation (rTMS) modulates evoked-gamma frequency oscillations in autism spectrum disorder (ASD)	43	Baruth JM (2010) [93]	J Neurother
5	Repetitive transcranial magnetic stimulation (rTMS) modulates event-related potential (ERP) indices of attention in autism	43	Casanova MF/Sokhadze E (2012) [94]	Transl Neurosci
6	Effects of low frequency repetitive transcranial magnetic stimulation (rTMS) on gamma frequency oscillations and event-related potentials during processing of illusory figures in autism	41	Sokhadze EM (2009) [95]	J Autism Dev Disord
7	Excitability changes induced in the human motor cortex by weak transcranial direct current stimulation	40	Nitsche MA (2000) [96]	J Physiol
8	A preliminary transcranial magnetic stimulation study of cortical inhibition and excitability in high-functioning autism and Asperger disorder	39	Enticott PG (2010) [79]	Dev Med Child Neurol
9	Prefrontal neuromodulation using rTMS improves error monitoring and correction function in autism	39	Sokhadze EM (2012) [50]	Appl Psychophysiol Biofeedback
10	Impaired motor facilitation during action observation in individuals with autism spectrum disorder	39	Théoret H (2005) [97]	Curr Biol

### Analysis of co-occurring keywords and clusters

**Fig. 6:**
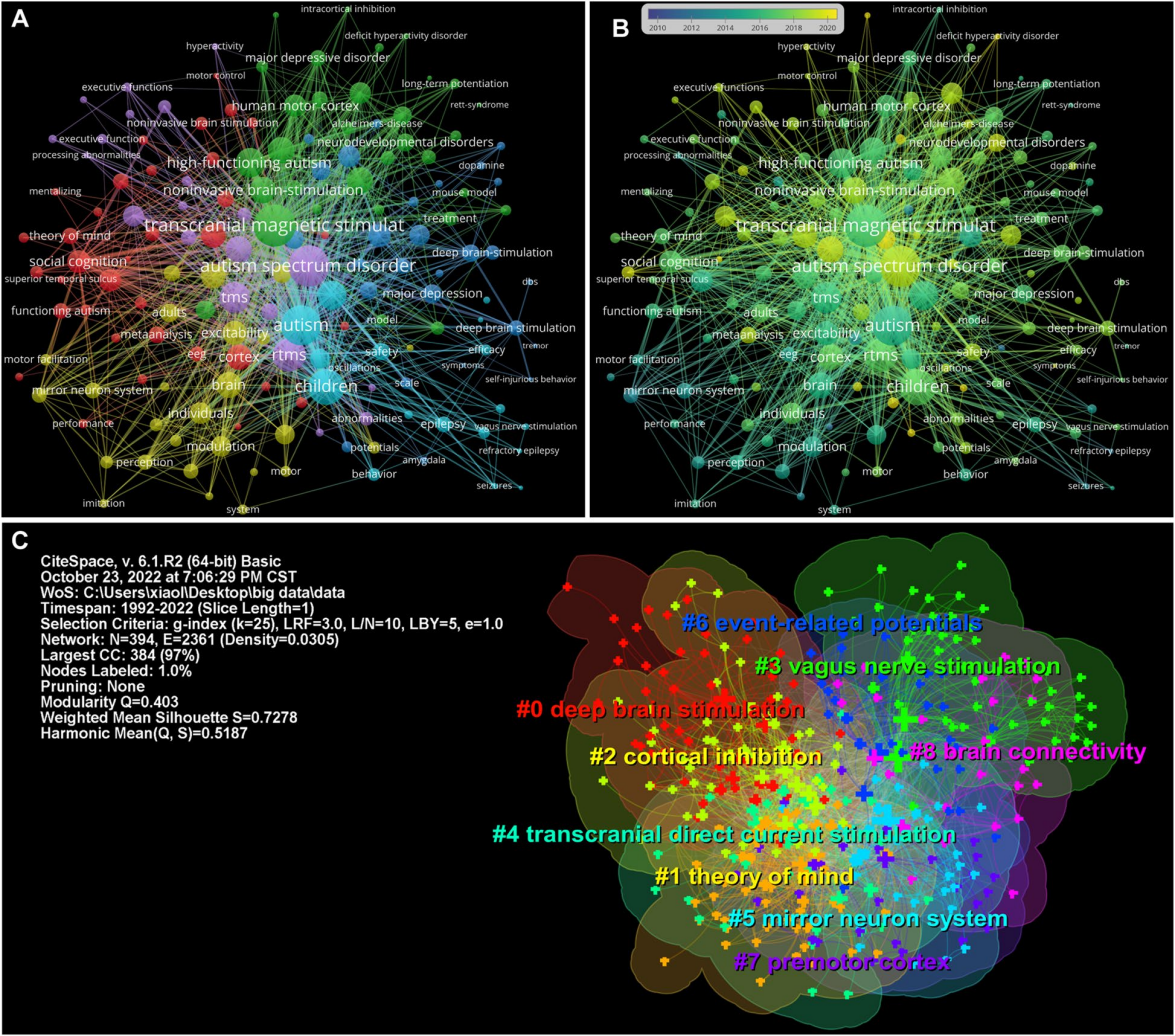
Analysis of co-occurring keywords. **A** Visualization of the co-occurring keywords network. **B** Activity analysis of the co-occurring keywords. **C** The cluster map of co-occurrence keywords

A total of 1,687 keywords were analyzed in this study, and the top 10 keywords with the strongest co-occurrence frequency are shown in Table 7, which is helpful for understanding the research hotspots in this field from 1992 to 2022. “Transcranial magnetic stimulation” is the keyword with the highest frequency of co-occurrence (130), followed by “Autism spectrum disorder” (120), and “Children” (63). For centrality, keywords with a centrality exceeding 0.1 are called “key nodes,” “Children” had the highest centrality (0.28), followed by “Autism spectrum disorder” (0.18) and “Transcranial magnetic stimulation” (0.15) (Table 7; Fig. 6A). Figure 6B shows that ASD has always been a hotspot in psychiatric disorders. In addition, studies on tDCS in ASD have become more frequent in recent years.

**Table 7:** Top 10 keywords related to neuromodulation techniques on ASD

Rank	Co-occurence	Centrality
1	Transcranial magnetic stimulation (130)	Children (0.28)
2	Autism spectrum disorder (120)	Autism spectrum disorder (0.18)
3	Children (63)	Transcranial magnetic stimulation (0.15)
4	Deep brain stimulation (47)	Deep brain stimulation (0.13)
5	high functioning autism (34)	Asperger syndrome (0.11)
6	rTMS (33)	Brain (0.11)
7	Asperger syndrome (32)	Autism (0.10)
8	Noninvasive brain stimulation (32)	Behavior (0.10)
9	Brain stimulation (29)	Excitability (0.10)
10	Double blind (29)	High functioning autism (0.09)

**Fig. 7:**
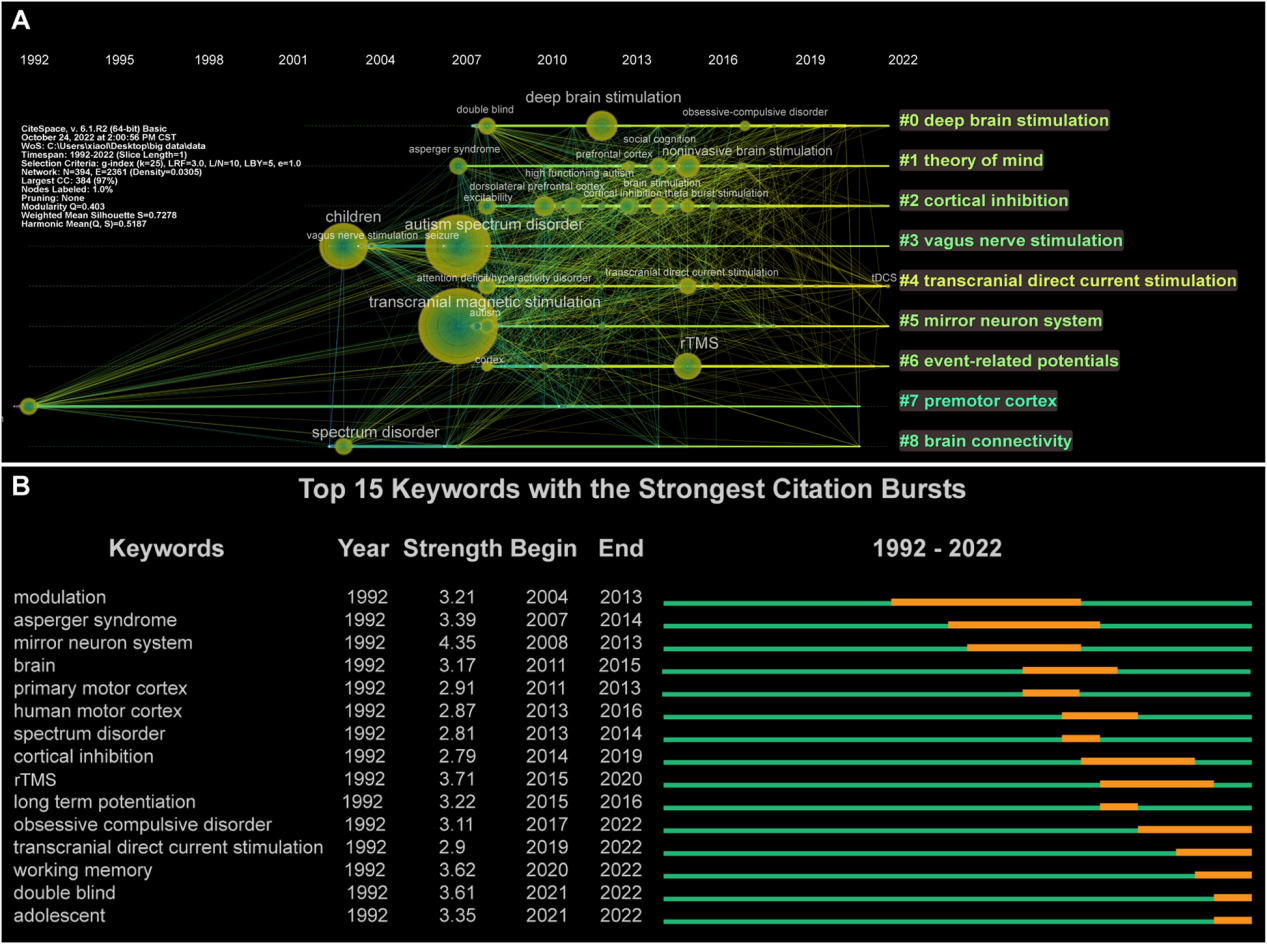
Analysis of keyword cluster timeline and burst keywords. **A** The cluster timeline view network of keywords from 1992 to 2022. The node size and color indicate the total number of references and individual time slices, respectively. Different colored lines indicate that two articles are co-cited in one article. The solid line and dotted line correspond to clustering topics representing hot and non-hot topics during the period, respectively. **B** The top 15 keywords with the strongest citation bursts

A cluster analysis of co-occurring keywords was performed using CiteSpace. In this study, the keywords were divided into nine clusters, and the cluster map classified the keywords according to their correlation. The data in the upper-left corner of Fig. 6C show two indicators: modularity and Mean Silhouette. When the modularity was greater than 0.3, the clustering structure was significant, and when the Mean Silhouette reached 0.7, the clustering results were convincing. In this study, the modularity was 0.403 and the Mean Silhouette was 0.728. Therefore, the results of the clustering structure were reasonable and convincing. “Deep brain stimulation,” “theory of mind,” “cortical inhibition,” “vagus nerve stimulation,” and “transcranial direct current stimulation” were the top 5 largest clusters (Table 8; Fig. 6C). To gain further insight into the evolution of these clusters, we visualized the keyword cluster timeline using CiteSpace. Figure 7A shows the timeline of the keyword cluster, which analyzes the occurrence and duration of the main keywords, which is helpful in understanding the research hotspots in this field across different time periods. After the first appearance of “vague nerve stimulation: in 2004, this term became highly related to “children,” “autism spectrum disorder,” “autism,” “seizure” and “social recognition,” which indicates that VNS may be a promising therapy for epileptic seizures and social dysfunction of autistic children [28, 98]. In addition, since its first appearance in 2007, “transcranial magnetic stimulation” has maintained a high correlation with “autism”, “Asperger syndrome”, “high-functioning autism”, “social recognition, and “cortical inhibition”, suggesting that TMS could improve the social defects of high-functioning autistic patients by altering cortical excitability [99, 100]. After the first appearance of “deep brain stimulation”, it has been widely associated with “depression” and “obsessive–compulsive disorder”, and clinical research on this topic has been common. Most studies on DBS treatment for ASD are in the preclinical stage [31–33]. It is worth mentioning that “transcranial direct current stimulation” maintains a high degree of contact with “children,” “autism,” “cortex,” “excitability” and “double blind,” and has been active in research in this field. In recent years, research has predominantly focused on randomized controlled trials for the treatment of autism [101].

**Table 8:** Top 9 clusters of keywords related to neuromodulation techniques on ASD

Cluster ID	Silhouette	Label		Included Keywords (Top 5)	Mean (Year)
0	0.645	Deep brain stimulation	Deep brain stimulation; Double blind; Major depressive disorder; Psychiatric disorder; Connectivity		2016
1	0.632	Theory of mind	Asperger syndrome; Noninvasive brain stimulation; Social cognition; Prefrontal cortex; Direct current stimulation		2015
2	0.718	Cortical inhibition	Excitability; High functioning autism; Human motor cortex; Dorsolateral prefrontal cortex; Brain stimulation		2014
3	0.817	Vagus nerve stimulation	Autism spectrum disorder; Children; Vagus nerve stimulation; Modulation; Behavior		2008
4	0.686	Transcranial direct current stimulation	Attention deficit/hyperactivity disorder; Adolescent; Transcranial direct current stimulation; Depression; Adult		2016
5	0.824	Mirror neuron system	Transcranial magnetic stimulation; Autism; Motor cortex; Activation; Corticospinal excitability		2012
6	0.782	Event-related potentials	Cortex; Attention; rTMS; Abnormality; Frequency		2013
7	0.791	Premotor cortex	Cruent; Perception; Asymmetry; Epilepsy; DC stimulation		2008
8	0.769	Brain connectivity	Spectrum disorder; Functional connectivity; EEG; Frontal cortex; Brain connectivity		2009

### Analysis of burst keywords

Finally, burst keywords were analyzed using CiteSpace. Burst keywords indicate that a keyword has received considerable attention over a period of time. We visualized the top 15 keywords with the strongest bursts (Fig. 7B), and found that “Modulation” was the keyword of the earliest burst, maintaining a high intensity outbreak in 2004–2013, followed by “Asperger syndrome” and “mirror neuron system”. Since 2011–2016, the keywords are mainly concentrated in terms related to the brain or cortex, such as “brain,” “primary motor cortex,” “cortical infection” and “long term potential,” suggesting that research during this period is related to the function of the cerebral cortex in ASD. “Obsessive–compulsive disorder,” “transcranial direct current stimulation” “working memory,” “double blind” and “adolescent” have been the most focused keywords in the last 5 years. It is worth noting that “transcranial direct current stimulation”, “double blind” and “adolescent” have been the most active burst keywords since 2019. In the past two years, clinical research on tDCS has mainly been carried out in children and adolescents with autism, and has played a positive role in the recovery of social functions [66, 68, 102]. This may reflect future research trends to a certain extent and may be related to the clinical trials in the treatment of adolescent psychiatric disorders by tDCS.

## Discussion

A previous bibliometric study found that research on ASD in the past 20 years mainly focused on its etiology, including brain structure, images, psychology, genes and animal models [103]. From 1998 to 2009, the classification and research of ASD and its subtypes have made great progress [104]. In particular, the Diagnostic and Statistical Manual of Mental Disorders (Fourth edition) (DSM-IV) use “pervasive developmental disorder” to name ASD, which including five subtypes: Rett Syndrome, Asperger Syndrome, Childhood Disintegrative Syndrome and Autistic Disorder. In addition, the etiology research of ASD is mainly focused on developmental neuroimaging and genetics during this period [101]. Many key brain regions have been proved to be related to the occurrence and development of autism, such as the medial prefrontal cortex, temporal-parietal junction [103], which provide potential options for the future treatment of ASD. From 2010 to 2018, with the promotion of the fifth edition of ASD diagnostic criteria in 2013, the research on ASD subtypes has been reduced to a certain extent, and chromosome and gene detection technologies have been widely used in ASD, which promoted the development of cytogenetic diagnostic tests for developmental delay/intellectual disability, ASD, or multiple congenital anomalies [101]. In addition, a large number of autism-related genes or risk genes have been found [104–106], such as PTCHD1 gene mutation on Xp22.11 [104]. However, some researchers believed that these gene mutations are lack of specificity and may widely exist in many mental diseases, so further exploration is needed [107]. In any case, the results of gene research provide a certain direction for the diagnosis and treatment of ASD or other mental diseases. In recent years, related research gradually turned to the comorbidities of ASD. ASD patients are often accompanied by comorbid symptoms such as anxiety, depression, attention deficit hyperactivity disorder and self-injurious behavior, etc., which bring serious burden to families and society. During the COVID-19 epidemic, home isolation may bring more negative effects to ASD children and their parents, such as more serious and frequent destructive behaviors in children and the depression and anxiety of parents. A large number of studies have confirmed that patients with ASD also show many digestive system symptoms, such as abdominal distension, abdominal pain and diarrhea, which may be related to disorders of intestinal flora [108–110]. Abnormal intestinal metabolites interfere with brain function and behavior through the microbiota, including metabonomic pathway [111] and vagal pathway [112]. The research on the treatment of ASD has been continuous in the past 20 years. The treatment of ASD is still based on education and behavior training, such as the applied behaviour analysis (ABA). However, in recent years, candidate drugs for the treatment of ASD have also brought hope. For example, the application of bumetanide, cannabis diphenol and oxytocin improves the severity of ASD symptoms and combined symptoms, including repeated patterns of behavior, interest or activity [113–115]. In addition, complementary and alternative treatments (CAT) has also received a lot of attention and has shown a positive role, such as dietary supplement and music therapy [116–118]. In the past 20 years, with the development of medical physics and medical physiology, based on the early research of etiology in ASD patients, neuromodulation techniques has come to the fore and has been widely used in preclinical and clinical research of treatment for ASD.

To the best of our knowledge, this is the first study to use VOSviewer and CiteSpace to review the progress of research on neuromodulation techniques as a treatment for ASD. To the best of our knowledge, this is the first study to use VOSviewer and CiteSpace to review the progress of research on neuromodulation techniques as a treatment for ASD. As such, we explore the characteristics of the publications from 1992 to October 2022, and our results reveal the current research focus and future prospects. Since the first study was published in 1992, the number of publications in this field has continued to grow. In particular, since 2014, research achievements in this field have surged, which may be due to the following factors: first, the diagnostic criteria of “Autism Spectrum Disorder” in the Diagnostic and Statistical Manual of Mental Disorders (fifth edition) (DSM-5) released in 2013 were more extensive, which may have increased the diagnostic rate of ASD [72, 73]. Second, this may be related to the release of the European Expert Group’s guidelines on the use of repeated TMS treatment in 2014, which has promoted research cooperation in this field [119]. The number of publications in 2020 declined slightly, which may be related to the reduction in international academic cooperation caused by the COVID-19 pandemic [120]. However, according to the current trend analysis, research on the treatment of autism using neuromodulation techniques will continue to show an overall growth trend in the next few years.

The authors of the publications published in the past 31 years covering Asia, Europe, North America, South America, and Oceania. The USA and Canada have become central members of a worldwide collaborative network, focusing on the role and mechanism of repetitive transcranial magnetic stimulation rTMS and tDCS in adult patients with autism in recent years [47, 121]. It is worth noting that, despite the existence of international cooperation, research in this field is mainly concentrated in developed or high-income countries, and African countries are not involved. Recent research has shown that the age-standardized prevalence of ASD in Africa is higher than the global average [2]. However, there are few studies in this field in Africa. The top three countries contributed 271 papers, accounting for 69.1% of all papers. The USA, which ranked first in the number of papers, contributed 170 papers. The top three institutions in terms of the number of papers contributed 64 papers, accounting for 16.8% of all papers. These results show that research in this field is unevenly distributed among countries or regions, and further promotion of participation and cooperation of institutions and countries is needed. The number of papers published and cited by the top 10 authors was 172 (43.9% of all papers) and 4720 (15.5% of the total papers cited), respectively. This means that most publications and influences are concentrated among a few co-authors, and most authors still lack a cooperative relationship. This may limit research progress on the treatment of ASD using neuromodulation techniques. The top 10 journals in terms of number of publications and citations were mainly involved in the fields of neuromodulation, neuroscience, and mental cognition, which showed that neuromodulation techniques have received extensive attention in neuropsychiatric disorders, especially in improving the psychological and cognitive impairment of patients with ASD [47, 48, 76, 83, 91].

In bibliometrics, keywords represent a high generalization of an article, whereas high-frequency keywords are often used to identify hot spots and frontiers in the research field. In this study, the top three keywords in terms of co-occurrence and centrality were “Children,” “Autism spectrum disorder” and “Transcranial magnetic stimulation,” which shows the importance and popularity of TMS in the treatment of autistic children. In the past 20 years, “children” has been a keyword with high co-occurrence frequency and representativeness, indicating that children are the key population in ASD research [1, 72, 122]. In recent years, the incidence of ASD in children has been rising, which may be related to the popularization of family health education concepts and the progress of overall medical diagnosis, early screening, and intervention measures [123, 124]. It is worth noting that in the past 40 years, our understanding of autism has significantly progressed, but the services of adults with autism still lags far behind those for children, and diagnosis and treatment may face greater challenges [125]. Peter G Enticott, McLeod Frampton Gwynette, Pushpal Desarkar, Stephanie H Ameis, and Douglas Teixeira Leffa. etal. have conducted significant research on adult autistic patients, suggesting that TMS and tDCS will bring clear benefits to their social functions and emotions, which may help promote the development of clinical treatment for adult autistic patients [76, 77, 92, 126, 127]. “Modulation” is the keyword with the highest burst intensity, and is also the core content of treatment in ASD. Previous studies have shown that mental disorders such as ASD may arise from an imbalance of E/I in the neural microcircuit, whereas the compensatory increase in the excitability of inhibitory cells partially alleviates the social defects caused by the increase in E/I balance [55, 128, 129]. Recent studies have shown that non-invasive neuromodulation techniques can regulate the distribution of neurotransmitters and metabolites in the brain [47, 48, 55], and improve the social and cognitive functions of patients with autism by altering the excitability of the cortex and neural circuits [39, 51, 79], which provides a theoretical basis for follow-up research in the treatment of ASD. The burst keywords in the most recent five years mainly focus on “obsessive–compulsive disorder,” “transcranial direct current stimulation,” “working memory,” “double blind” and “adolescent”. Although in our analysis, these were five independent high-frequency keywords, in the actual publications, we found that these keywords often appeared together. Previous studies have shown that ASD and obsessive–compulsive disorder (OCD) are highly comorbid, and that there may be a pathophysiological basis for comorbidity between them [130, 131]. A prior study showed that 25% of young people with OCD were diagnosed with ASD, and 5% of young people with ASD were diagnosed with OCD [132]. ASD combined with OCD is associated with more overall dysfunction, which is far more difficult to treat than a single disease [133, 134]. A recent study showed that DBS in the ventral forelimb of the internal capsule or medial forebrain bundle could reduce the symptoms of obsessive–compulsive behavior and depression, which indicates that neuromodulation techniques may be an effective intervention in the comorbidity of ASD, but further exploration is needed [135]. In addition, we found that some recent clinical studies predominantly focused on the positive effects of tDCS in adolescents with ASD, including social cognition and emotional behavior [66, 68, 102]. Working memory is often impaired in ASD, which may be the basis of the core defects of cognitive and social functions [136]. These findings suggest that tDCS is a simple and well-tolerated adjuvant therapy that can improve the quality of life and autonomy of patients with ASD. Based on our analysis and the above literature review, the five keywords “obsessive–compulsive disorder,” “transcranial direct current stimulation,” “working memory,” “double blind” and “adolescent” have gradually come to reflect hot spots and fronts in the field of neuromodulation techniques in the treatment of ASD.

### Limitations

This study has several limitations. First, as the data are only from the WoS database, the analysis may not be sufficiently comprehensive. Second, our search was limited to literature published in English, which makes the analysis incomplete to a certain extent. Although only 11 articles written in non-English languages were excluded, these still had reference values and significance. In addition, although VOSviewer and CiteSpace are professional bibliometric analysis software tools that allow objective analysis, different researchers may have different perspectives on the same content; therefore, their bias is unavoidable. Finally, the text content of some pictures displayed using these software programs is incomplete; although this does not affect the understanding of the literature, these software programs need to be further improved.

## Conclusion

In conclusion, the etiology of ASD is complex, and its treatment is the most challenging topic in clinical and basic research. This study investigated the trends in the development of neuromodulation techniques for the treatment of ASD from 1992 to 2022 using bibliometric analysis. Based on 392 articles obtained from WoS, we identified important publications, authors, journals, institutions, and countries, and further analyzed the relationship among them, to reveal the research status of neuromodulation techniques in the treatment of ASD, as well as the hotspots and research fronts. Treatment of autism using neuromodulation techniques is a relatively new field. With the promotion of interdisciplinary and technological progress, the treatment mode of neuromodulation techniques will continue to improve, and targeted indications will gradually increase. tDCS is increasingly used in the clinical research of children and adolescents with ASD, which may become a potential choice for the treatment of autism. Improving the social ability and symptoms of comorbidities of these ASD patients, such as obsessive–compulsive disorder and epilepsy, will also continue to be the focus of global research.

## Supplementary Information

**Additional file 1.**

**Additional file 2.**

## Data Availability

The original data from the current study are available from the corresponding author upon reasonable request.

## Abbreviations

ASD: Autism spectrum disorder
WoS: The web of science
TMS: Transcranial magnetic stimulation
tDCS: Transcranial direct current stimulation
DBS: Deep brain stimulation
VPA: Valproate acid
VNS: Vagus nerve stimulation
FDA: Food and drug administration
EEG: Electroencephalogram
DSM-5: Diagnostic and statistical manual of mental disorders (Fifth Edition)
ACNP: American college of neuropsychopharmacology
SOBP: Society of biological psychiatry
ECNP: Congress of the European college of neuropsychopharmacology
COVID-19: Corona virus disease 2019
rTMS: Repetitive transcranial magnetic stimulation
OCD: Obsessive–compulsive disorder
