# Supplementary figures and images for: A bibliometric analysis of global research status and trends in neuromodulation techniques in the treatment of autism spectrum disorder
Source: BMC Psychiatry. 2023 Mar 20;23:183. doi: 10.1186/s12888-023-04666-3 (PMC10026211; doi:10.1186/s12888-023-04666-3)

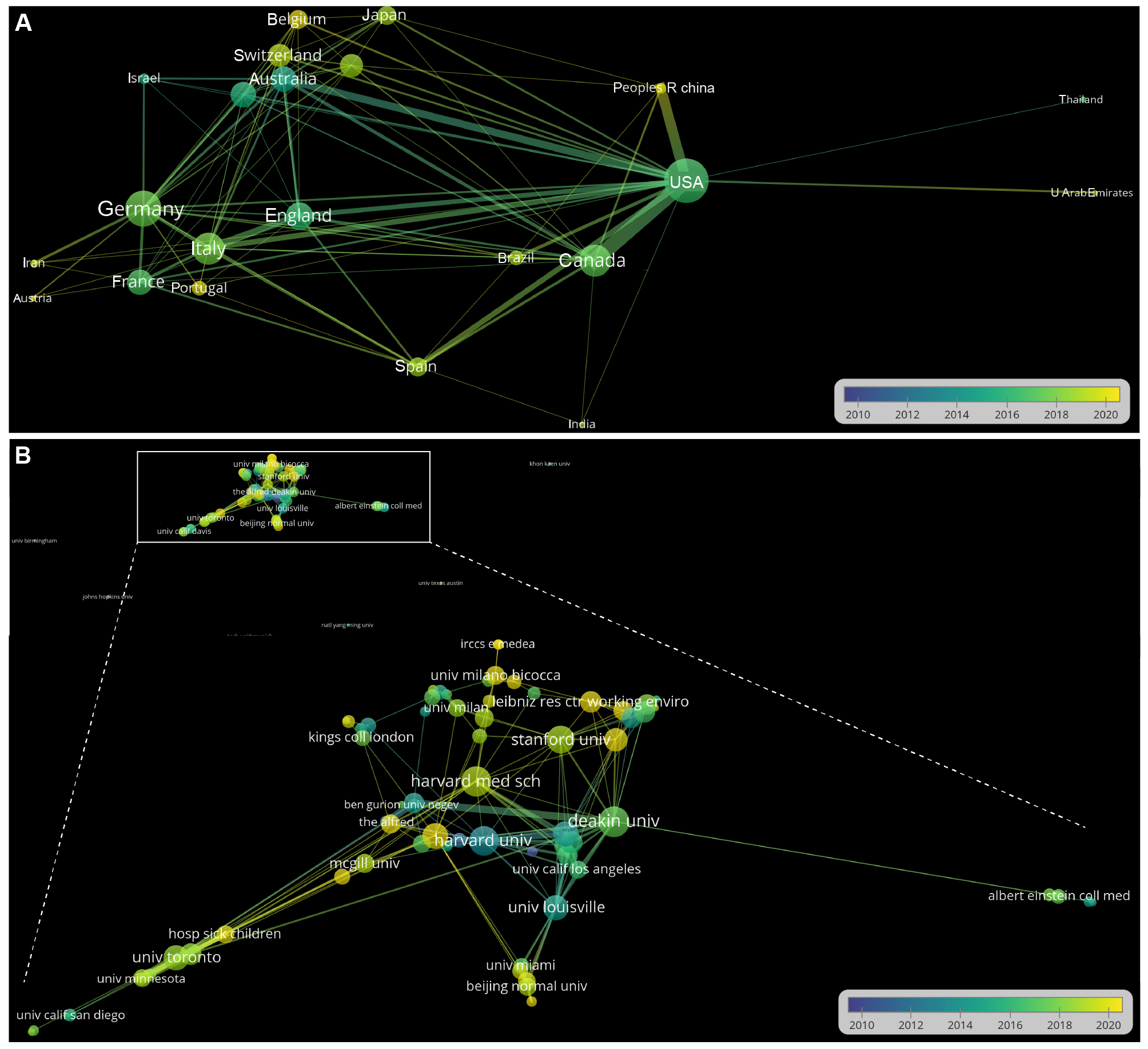

Supplement: Supplementary file 1 — Additional file 1. [file 12888_2023_4666_MOESM1_ESM.zip › Supplementary Figure 1.tif]

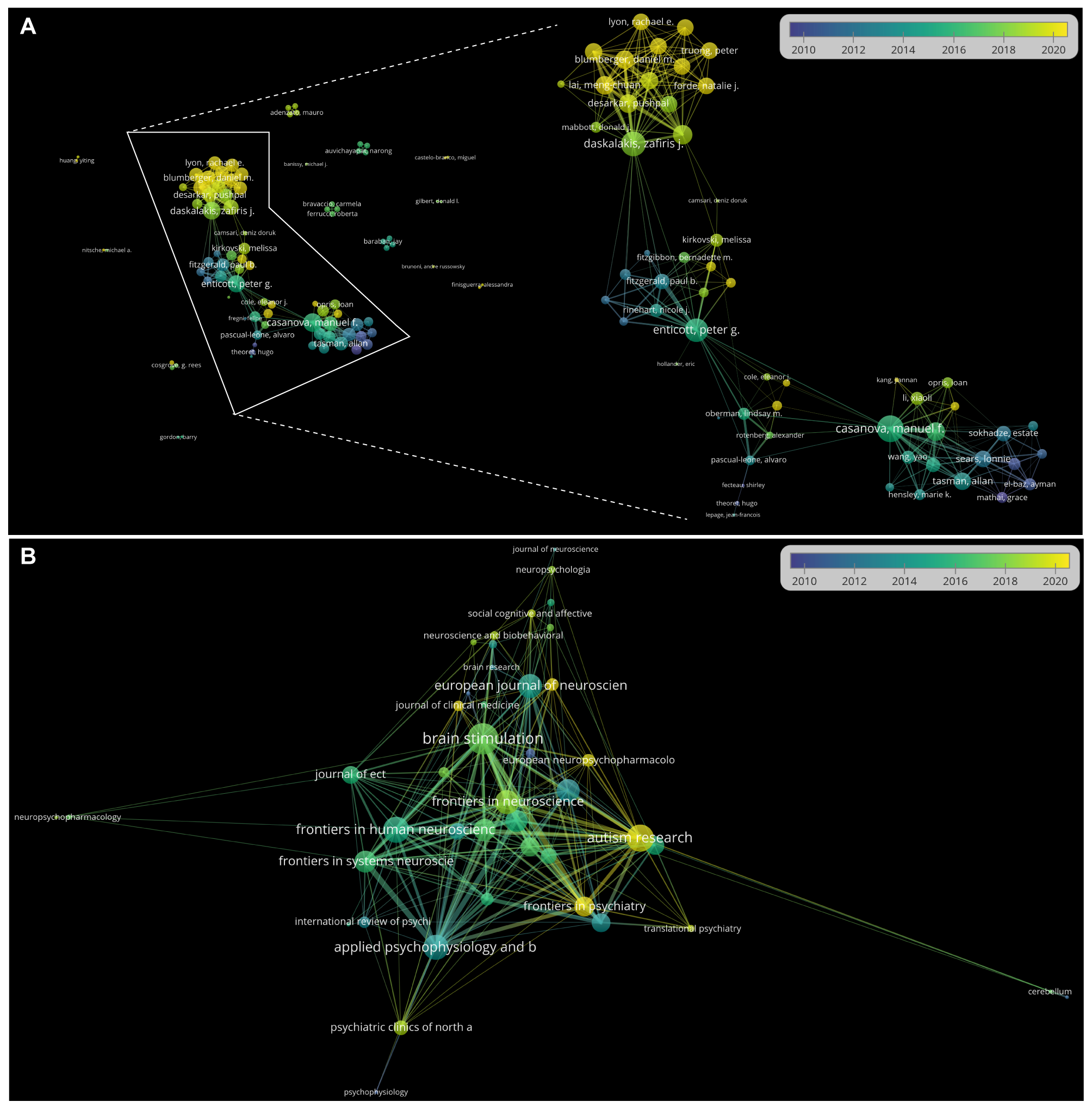

Supplement: Supplementary file 2 — Additional file 2. [file 12888_2023_4666_MOESM2_ESM.zip › Supplementary Figure 2.tif]
